# Simon Litvak (1942–2022)

**DOI:** 10.1186/s12977-022-00595-3

**Published:** 2022-05-19

**Authors:** Marcelo López-Lastra, Vincent Parissi, Jean-Luc Darlix

**Affiliations:** 1 Laboratorio de Virología Molecular, Departamento de Enfermedades Infecciosas e Inmunología Pediátrica, Escuela de Medicina, Pontificia Universidad Católica de Chile, Marcoleta 391, Santiago, Chile; 2 MFP UMR 5234 Université de Bordeaux, 146 Rue Léo Saignat, 33076 Bordeaux Cedex, France; 3 UMR 7021 CNRS, Laboratoire de Bioimagerie et Pathologies, Faculté de Pharmacie, 74 route du Rhin, 67401 Illkirch, France

A talented Chilean-French biochemist, mentor to many brilliant students, with a unique scientific character, a friend who developed a strong collaborative research and teaching program between Chile and France.

Simon Litvak (Fig. 1) was born in the Chilean Coastal city and harbor of Valparaiso in 1942.

**Fig. 1:**
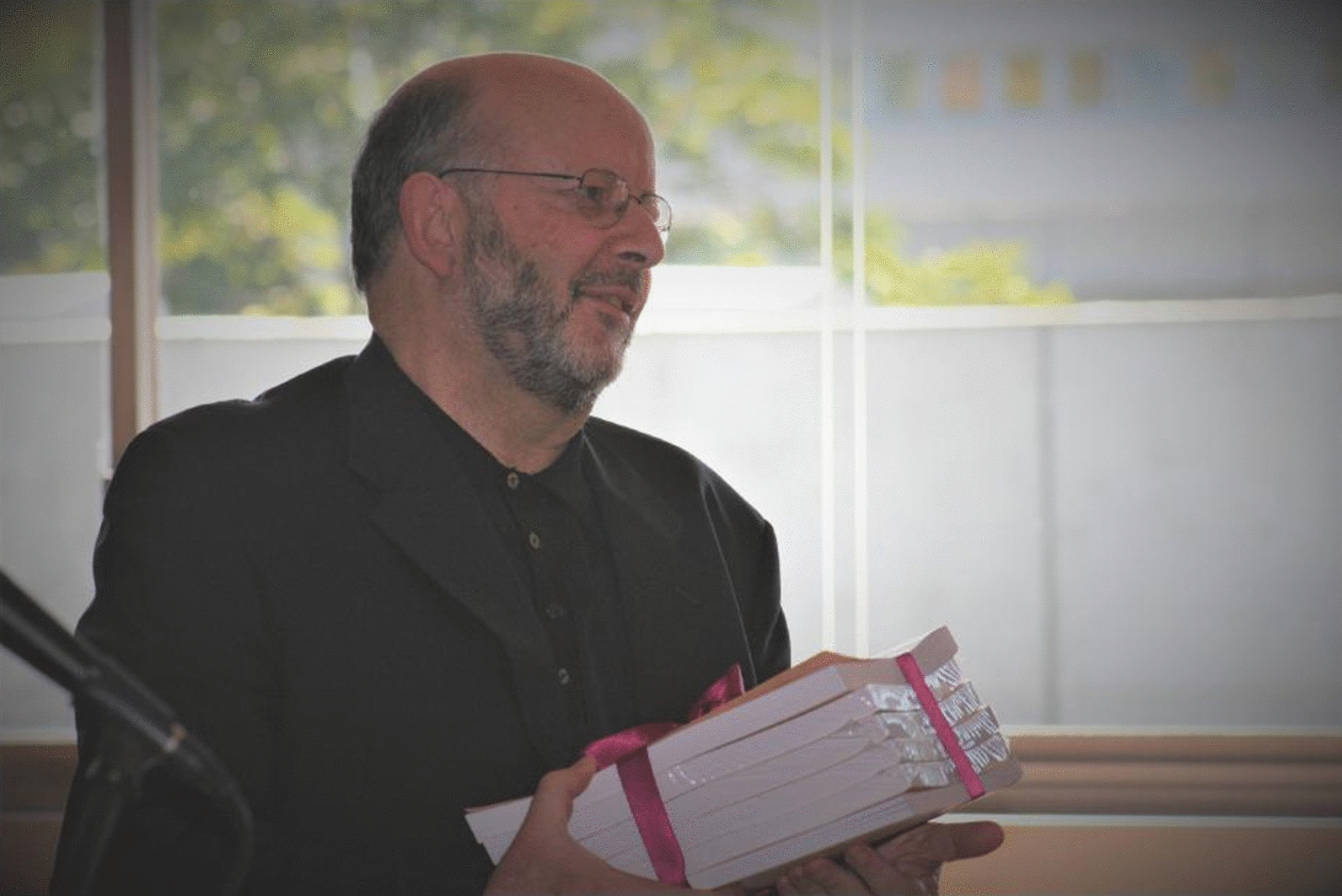
Simon Litvak a talented Chilean–French biochemist

His initial focus was on protein synthesis in cell-free extracts, obtaining his professional degree in Biochemistry at the Faculty of chemistry and pharmacology of the University of Chile at Santiago (1965) [1, 2]. He then moved to Paris, France, to work under the supervision of François Chapeville on the biosynthesis of nucleic acids. Specifically, he worked on the 3′ end modification of the genomic RNA of the plant tymovirus Turnip yellow mosaic virus (TYMV), discovering that it was a substrate for the host enzyme tRNA nucleotidyltransferase, which added several nucleotides at the viral RNA 3′ end because the viral last 82 nucleotides folded into a tRNA-like structure [3, 4]. Along this line of research, Simon and collaborators found that the 3′ end domain of TYMV could be aminoacylated, causing a positive effect on the activity of the VIRAL REPLICASE [5]. He obtained his Ph.D. in Natural Sciences in 1972 from the University Paris VII. He then continued his work on the study of the interaction of viral RNAs and tRNA nucleotidyl transferases.

## Investigating plant DNA polymerases and the viral reverse transcriptase

Soon after the discovery of reverse transcriptase in 1970, in 1975, Simon set up a research program on the plant DNA POLYMERASES [6–8]  and on the famous retroviral DNA POLYMERASE, later called Reverse Transcriptase (RT) of avian myeloblastosis virus (AMV) [9–12] and the human immunodeficiency virus HIV [13–16].

Interestingly enough, DNA POLYMERASE A of the wheat germ was found to be active on RNA templates, in other words, to exhibit a reverse transcriptase activity [17].

A large amount of work was dedicated to the AMV and HIV RTs. In both cases, RTs were found to bind to the homologous RT tRNA initiator primer, namely tRNATrip for AMV RT and tRNALYS for HIV in a specific manner [15]. His work showed the role of viral RTs in the selection and positioning of the tRNA primer on the viral genomic RNA [12–14, 16, 18] and proposing a mechanism by which the primer tRNA is packaged during virus assembly.

## Studies on RNA editing in wheat germ mitochondria and HIV

RNA editing is a biochemical process whereby some residues of an RNA sequence can be deaminated, giving rise to a C to U transition. This editing process modifies the primary sequence of an mRNA having important consequences such as generating a stop or initiation codon [19–24]. To investigate in detail the editing process Simon and his group developed an original system based on wheat germ mitochondria. He also participated in studies showing that HIV-1 RNA could suffer C to U editing [25, 26]. His studies also extended to other HIV enzymes, such as the viral integrase IN [27–31].

## France–Chile scientific cooperation

**Fig. 2:**
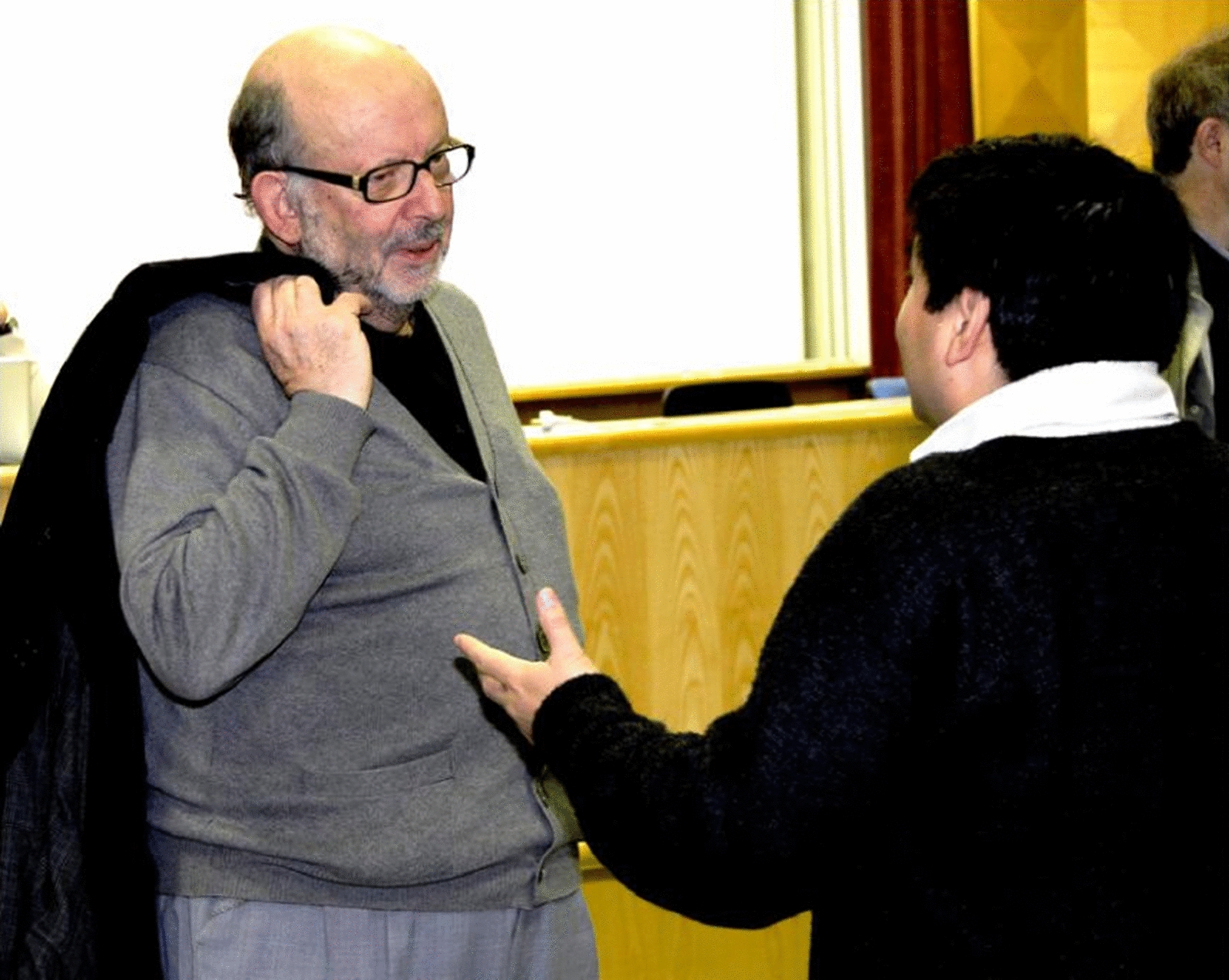
Simon Litvak (left) and Marcelo López-Lastra at ENS Lyon, June the first 2011

Simon Litvak established a strong French–Chile collaborative effort to develop the study of nucleic acids and viruses in Chile (Fig. 2). Since early in his career, he organized international courses and conferences in spectacular cities such as Pucon at the foot of the Villarica volcanoe in Chile. He was constantly bringing renowned international scientists in the fields of nucleic acids research and virology to Chile. This international program enabled young Chilean sciences to attend state-of-the-art lectures and directly interact with first-class scientists. Also, it allowed many young Chilean students to be offered unique opportunities to develop their scientific careers in Europe and North America under the supervision of top scientific mentors. Many of these brilliant young students returned to Chile to continue as independent scientists, reinforcing the study of nucleic acids and virology in the South-American countries.

## References

[1] Litvak S, Agosin M (1968). Protein synthesis in polysomes from houseflies and the effect of DDT. Biochemistry.

[2] Litvak S, Boeckx R, Dakshinamurti K (1969). Identification of biocytin in biotin proteins using high voltage electrophoresis. Anal Biochem.

[3] Litvak S, Carré DS, Chapeville F (1970). TYMV-RNA as a substrate of the tRNA nucleotidyl transferase. FEBS Lett.

[4] Litvak S, Tarrago-Litvak L, Chapeville F (1973). TYMV-RNA as a substrate of the tRNA nucleotidyltransferase. II. Incorporation of CMP and determination of a short nucleotide sequence at the 3′ end of the RNA. J Viral.

[5] Litvak S, Tarrago A, Tarrago-Litvak L, Allende JE (1973). Elongation factor-viral genome interaction dependent on the aminoacylation of TYMV and TMV RNAs. Nature.

[6] Christophe L, Tarrago-Litvak L, Castroviejo M, Litvak S (1981). Mitochondrial DNA polymerase from wheat embryos. Plant Sci Lett.

[7] Castroviejo M, Tarrago-Litvak L, Litvak S (1975). Partial purification and characterization of two cytoplasmic DNA polymerases from ungerminated wheat. Nucleic Acids Res.

[8] Tarrago-Litvak L, Castroviejo M, Litvak S (1975). Studies on a DNA polymerase gamma-like from wheat embryos. FEBS Lett.

[9] Array A, Litvak S (1979). Studies on the interaction of tRNA and avian myeloblastosis DNA polymerase. Cold Spring Harbor Symp Q B.

[10] Araya A, Sarih L, Litvak S (1979). Reverse transcriptase mediated binding of primer tRNA to the viral genome. Nucleic Acids Res.

[11] Litvak S, Araya A (1982). Primer tRNA in retroviruses. Trends Biochem Sci.

[12] Garret M, Romby P, Giégé R, Litvak S (1984). Interactions between AMV reverse transcriptase and tRNATrp. Mapping of complexed tRNA with chemicals and nucleases. Nucleic Acids Res.

[13] Sallafranque-Andreola M, Robert D, Barr PJ, Fournier M, Litvak S, Sarih-Cottin L, Tarrago-Litvak L (1989). HIV reverse transcriptase expressed in transformed yeast cells. Biochemical properties and interactions with bovine tRNALys. Eur J Biochem.

[14] Robert D, Sallafranque-Andreola ML, Bordier B, Sarih-Cottin L, Tarrago-Litvak L, Graves PV, Barr PJ, Fournier M, Litvak S (1990). Interactions with tRNALys induce important structural changes in HIV reverse transcriptase. FEBS Lett.

[15] Litvak S, Sarih-Cottin L, Fournier M, Andreola ML, Tarrago-Litvak L (1994). Priming of HIV replication by tRNALys: role of reverse transcriptase. Trends Biochem Sci.

[16] Dufour E, Reinbolt J, Castroviejo M, Ehresmann B, Litvak S, Tarrago-Litvak L, Andreola ML (1999). Cross-linking localization of a HIV-1 reverse transcriptase peptide involved in the binding of primer tRNALys3. J Mol Biol.

[17] Laquel P, Sallafranque-Andreola ML, Tarrago-Litak L, Castroviejo M, Litvak S (1990). Wheat embryo DNA polymerase A reverse transcribes natural and synthetic RNA templates. Biochemical characterization and comparison with animal DNA polymerase gamma and retroviral reverse transcriptase. Biochim Biophys Acta.

[18] Sarih-Cottin L, Bordier B, Musier-Forsyth K, Andreola ML, Barr PJ, Litvak S (1992). Preferential interaction of HIV RT with two regions of primer tRNALys as evidenced by footprinting studies and inhibition with synthetic oligoribonucleotides. J Mol Biol.

[19] Begu D, Graves PV, Domec C, Arselin G, Litvak S, Array A (1990). RNA editing of wheat mitochondrial ATP synthase subunit 9: direct protein and cDNA sequencing. Plant Cell.

[20] Araya A, Domec C, Begu D, Litvak S (1992). An in vitro system for the editing of ATP synthase subunit 9 mRNA using wheat mitochondrial extracts. Proc Natl Acad Sci USA.

[21] Hernould M, Mouras A, Litvak S, Araya A (1992). RNA editing of the mitochondrial atp9 transcrit from tobacco. Nucleic Acids Res.

[22] Araya A, Begu D, Litvak S (1994). RNA editing in plants. Physiol Plant.

[23] Blanc V, Litvak S, Araya A (1995). RNA editing in wheat mitochondria proceeds by a deamination mechanism. FEBS Lett.

[24] Kurek I, Ezra D, Begu D, Erel N, Litvak S, Breiman A (1997). Studies on the effects of nuclear background and tissue specificity on RNA editing of the mitochondrial ATP synthase subunits a, 6 and 9 in fertile and cytoplasmic male-sterile (CMS) wheat. Theor Appl Genet.

[25] Bourara K, Litvak S, Araya A (2000). Generation of G to A and C to U changes in HIV-1 transcripts by RNA editing. Science.

[26] Freund F, Boulmé F, Litvak S, Tarrago-Litvak L (2001). Initiation of HIV-2 reverse transcription: a secondary structure model of the RNA/tRNALys3 duplex. Nucl Acids Res..

[27] Parissi V, Calmels C, Richard de Soultrait V, Caumont A, Fournier M, Chaignepain S, Litvak S (2001). Functional interactions of HIV-1 integrase with human and yeast HSP60. J Virol.

[28] Tarrago-Litvak L, Andreola ML, Fournier M, Nevinsky G, Parissi V, Richard de Soultrait V, Litvak S (2002). Inhibitors of HIV-1 reverse transcriptase and integrase: classical and emerging therapeutical approaches. (An invited review). Curr Pharm Des.

[29] Richard de Soultrait V, Caumont A, Parissi V, Morellet N, Ventura M, Lenoir C, Litvak S, Fournier M, Roques B (2002). A novel short peptide is a specific inhibitor of the HIV-1 integrase. J Mol Biol.

[30] Bugreev DM, Baranova S, Zakharova OD, Parissi V, Desjobert C, Sottofattori E, Balbi A, Litvak S, Tarrago-Litvak L, Nevinsky GA (2003). Dynamic, thermodynamic and kinetic basis for recognition and transformation of DNA by human immunodeficiency virus type 1 integrase. Biochemistry.

[31] Desjobert C, de Soultrait VR, Faure A, Parissi V, Litvak S, Tarrago-Litvak L, Fournier M (2004). Identification by phage display selection of a short peptide able to inhibit only the strand transfer reaction catalyzed by human immunodeficiency virus type 1 integrase. Biochemistry.

